# Supplementary material for: The interactome of intact mitochondria by cross-linking mass spectrometry provides evidence for coexisting respiratory supercomplexes
Source: Mol Cell Proteomics. 2017 Dec 8;17(2):216–32. doi: 10.1074/mcp.RA117.000470 (PMC5795388; doi:10.1074/mcp.RA117.000470)
Supplement: Supplemental Data [file supp_RA117.000470_133922_0_supp_23978_fzffwf.zip › spectra_annotation/mito_DR_spectra_annotation/333-1-5-1-8-1.pdf]

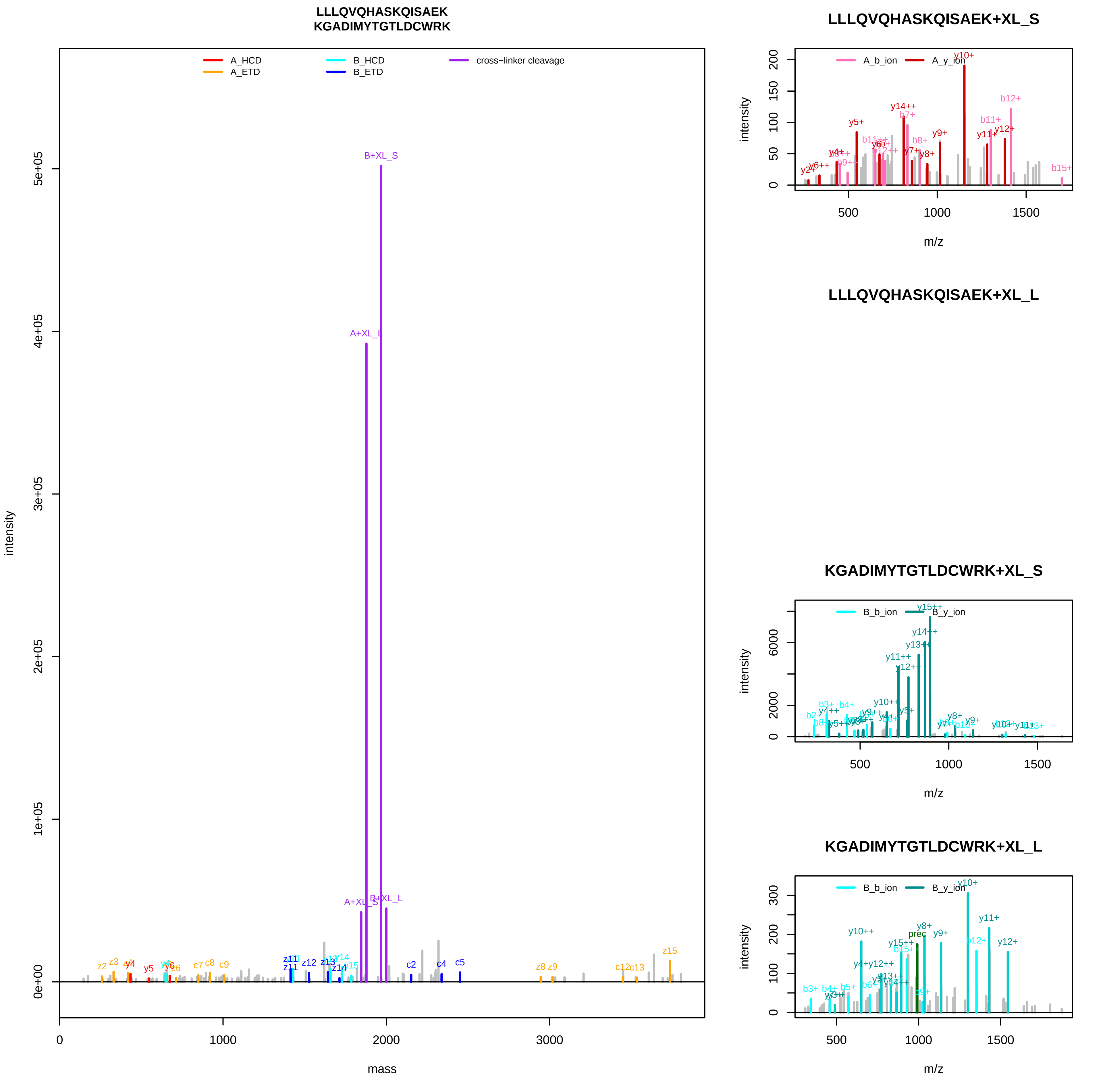

LLLQVQHASKQISAEK
KGADIMYTGTLDCWRK
A_HCD
A_ETD
B_HCD
B_ETD
cross−linker cleavage
B+XL_S
A+XL_L
A+XL_S
B+XL_L
intensity
mass
0e+00
1e+05
2e+05
3e+05
4e+05
5e+05
0
1000
2000
3000
LLLQVQHASKQISAEK+XL_S
A_b_ion
A_y_ion
y10+
b12+
y14++
b7+
y5+
b11+
y12+
y11+
y9+
b8+
y6+
y7+
y8+
y4+
y6++
y2+
b15+
intensity
m/z
LLLQVQHASKQISAEK+XL_L
KGADIMYTGTLDCWRK+XL_S
B_b_ion
B_y_ion
y15++
y14++
y13++
y11++
y12++
y10++
intensity
m/z
KGADIMYTGTLDCWRK+XL_L
B_b_ion
B_y_ion
y10+
y11+
y10++
y8+
prec
y9+
y12+
b12+
y15++
intensity
m/z
